# The Nanocarrier Landscape—Evaluating Key Drug Delivery Vehicles and Their Capabilities: A Translational Perspective

**DOI:** 10.1021/acsami.5c07366

**Published:** 2025-06-17

**Authors:** Fahd Khalid-Salako, Soodeh Salimi Khaligh, Farzaneh Fathi, Osman C. Demirci, Nazlı Öncer, Hasan Kurt, Meral Yüce

**Affiliations:** † SUNUM Nanotechnology Research and Application Centre, 52991 Sabanci University, Istanbul 34956, Türkiye; ‡ Pharmaceutical Sciences Research Center, Ardabil University of Medical Sciences, Ardabil 56131-56491, Iran; § Gebze Technical University, Department of Environmental Engineering, Gebze, Kocaeli 41400, Türkiye; ∥ Department of Bioengineering, Royal School of Mines, 4615 Imperial College London, London SW7 2AZ, U.K.

**Keywords:** nanocarriers, nanoparticle drug delivery, toxicity, biocompatibility, personalized nanomedicine, theranostics

## Abstract

The field of nanomedicine is currently in a revolutionary phase, propelled by the significant potential of nanoparticles, which offer several advantages over traditional drug delivery systems. The purpose of this paper is to aggregate contemporary knowledge of nanoparticles developed and applied in drug delivery across major disease classes. Accordingly, we offer, through a thorough search of the literature, a comprehensive overview of the prevalent nanoparticles used in drug delivery systems, covering polymeric, lipid-based, inorganic, and carbon-based nanoparticles, and discuss their advantages and limitations. This work primarily focuses on studies published in the last 5 years, aiming to provide an up-to-date assessment of the critical nanoparticles in drug delivery. Narratively, we synthesize a comprehensive overview of the state-of-the-art in nanocarrier technology, providing in-depth insights into the key nanoparticle types presented in the contemporary literature, their fundamental benefits, potential clinical applications, and limitations impeding their development and adoption. We note that there are gaps and opportunities for concerted efforts focused on developing biocompatible and biodegradable nanoparticles, establishing scalable and cost-effective manufacturing processes, and addressing regulatory challenges associated with nanoparticle-based drug delivery systems. These challenges persist despite the immense translational success of nanoparticle-based drug delivery systems and necessitate continued interdisciplinary research and cross-industry collaboration among scientists, clinicians, and regulatory bodies.

## Graphical abstract

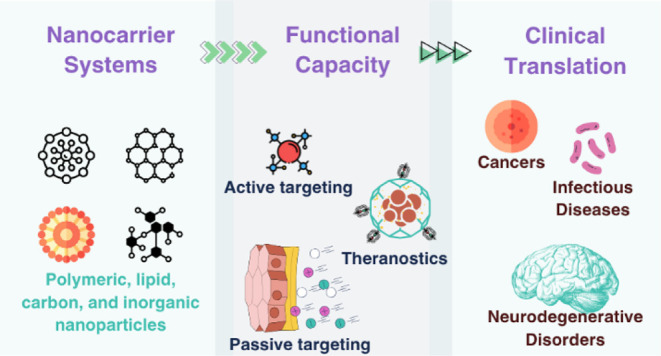

## 1 Introduction

The physicochemical properties of materials constituting conventional drug delivery systems have significant implications for drug release profiles and have been demonstrated to cause bioavailability constraints and inconsistent plasma levels, leading to subpar clinical responses and, ultimately, adverse drug reactions.^1^ From a clinical or translational viewpoint, the insufficiency of traditional delivery systems arises from unfavorable material properties that impair solubility and bioavailability and have significant ramifications for clinical outcomes and patients’ quality of life. It is necessary to overcome these limitations within the pharmaceutical industry, and one of the approaches that have been recently explored is nanoparticle-based drug delivery systems. These systems are based on nanocarriers, possessing unique features that enhance biodistribution, stability, solubility profiles, and other pharmacokinetic parameters, ultimately reducing toxicity, with the added possibility of more precisely controlled cargo delivery.^2^ Leveraging these properties, drugs encapsulated within or conjugated with nanoparticles can be delivered in a manner that enhances therapeutic outcomes and reduces adverse effects in practical terms.

Fundamentally, some of the most explored benefits of nanocarriers include enhancement of drug pharmacokinetics; targeted and controlled delivery; and theranostic functionalizations. In terms of pharmacokinetic enhancement, nanocarrier drug delivery systems offer better solubility and bioavailability profiles, overcoming the physicochemical limitations of several active pharmaceutical ingredients (APIs). As has been discussed in recent literature, nanoparticles effectively achieve better aqueous solubility of hydrophobic APIs.^3,4^ This is especially important for less soluble drugs such as hydrocortisone, the properties of which can be enhanced by encapsulation in a chitosan-coated magnetic core–shell nanocarrier.^5^ In a similar vein, Uhl et al. functionalized drug-loaded polylactic acid (PLA) nanoparticles with a cyclic cell-penetrating peptide for higher oral liraglutide stability and bioavailability.^6^ In effect, enhancing solubility and bioavailability using nanocarriers results in better drug absorption, distribution, and therapeutic efficacy, while reducing reliance on organic solvents or surfactants with safety concerns.^7^ It is this pharmacokinetic enhancement capability of nanocarriers that has largely driven their adoption for APIs that are unstable and difficult to store, handle, and administer.^8–10^ As recently noted, nanoparticles provide physical barriers, controlled release, minimized exposure to degradation, and surface modification potential that can be leveraged to enhance the stability of unstable biologic agents.^11^ While this protective property depends on the nanoparticle composition and drug-loading efficiency, the general propensity of nanocarriers to improve the shelf life of unstable drugs has, over time, proven valuable in protein and gene therapy, among other biologic agents.^12–17^

A quick glance through recent literature shows that some of the most promising applications of nanocarriers are targeted, controlled delivery and functionalizations for theranostic applications. They have also been discussed in recent literature for the delivery of natural products, not only enhancing their bioavailability and pharmacokinetics but also providing much-needed dosage standardization and social acceptance.^18^ By their nature, nanocarriers can be tailored during synthesis and functionalization for specific release behaviors that enhance therapeutic efficacy by maintaining drug concentrations within the therapeutic window for an extended period. The impact of these possibilities is wide-reaching and demonstrable in both clinical and humanistic outcomes. Advanced nanocarrier systems reduce the need for frequent dosing, improving patient compliance and quality of life. Nanocarriers also provide specific properties that solve very peculiar disease management challenges, such as antimicrobial resistance in infectious diseases and biodistribution in neurological diseases.^19,20^

There is a plethora of strategies employed to tune release kinetics from nanocarriers, including modifying the nanoparticle composition, size, and surface properties and incorporating stimuli-responsive principles that trigger drug release in response to specific environmental cues such as pH, temperature, or enzymatic activity.^21^ Targeted release minimizes off-target effects and ensures cargo delivery only under the right pathophysiological circumstances. Polymer-based nanoparticles, liposomes, and inorganic and carbon nanotubes are some of the nanocarriers that have been developed to respond to extrinsic actuators of cargo release.^4^ Moreover, nanocarriers can be engineered to selectively accumulate in target sites through passive targeting, which exploits certain pathophysiological characteristics (hyperacidity and hyperthermia in tumors, for example), or active targeting, wherein a ligand with high affinity and specificity for a target protein is attached to the nanoparticle surface, improving drug internalization and increasing the local concentration of the therapeutic payload.^22–25^

The theranostic potential of nanocarriers makes them advantageous over conventional diagnostic and therapeutic methods.^26^ Nanocarriers incorporating imaging agents, such as fluorescent dyes, radionuclides, or contrast agents, can provide real-time visualization and tracking of drug biodistribution and accumulation *in vivo*. One notable example of a theranostic nanocarrier is anticancer drug-loaded iron oxide nanoparticles functionalized with targeting ligands and magnetic resonance imaging (MRI) contrast agents, concurrently allowing targeted drug delivery and MRI-based real-time monitoring of *in vivo* drug accumulation for personalized treatment strategies.^27^ Similarly, gold nanoparticles are deployed in photothermal therapy (PTT), done by converting absorbed light into heat by a nonradiative process.^28,29^ Combining this capability with near-infrared photothermal imaging, Guan et al. demonstrated that clustered gold nanoparticles showed theranostic dual capability in human prostate cancer cells with high efficiency and selectivity.^30^

Some of the nanocarrier designs reported in the literature for targeted and controlled drug delivery as well as theranostic applications are illustrated in Figure 1.

**Figure 1:**
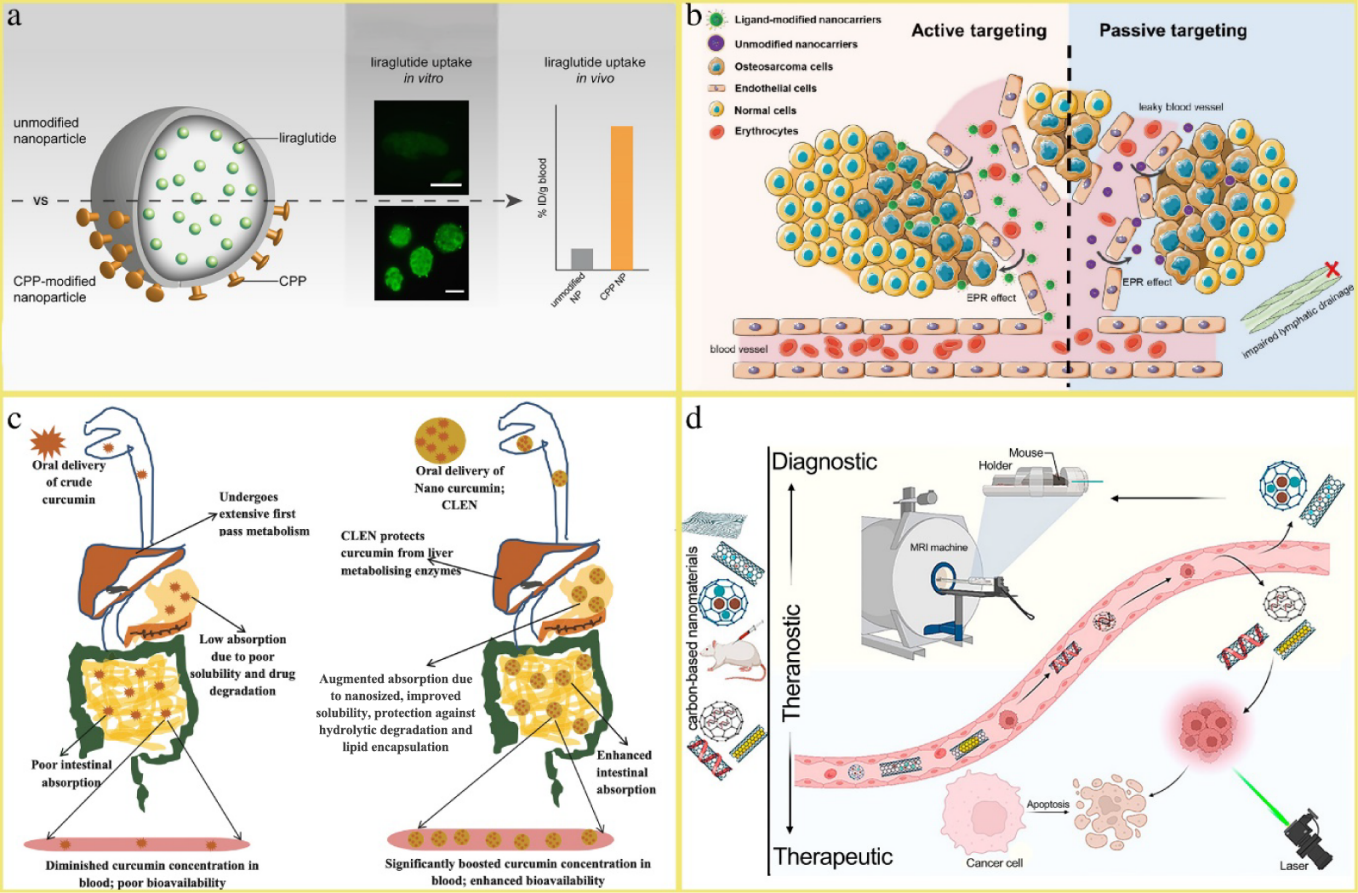
Schematic illustrations of a) Enhancement of the oral uptake of liraglutide through polylactide acid (PLA) nanoparticles. Reproduced with permission from ref 6. Copyright 2019 Elsevier Inc.; b) Active targeting and passive targeting of nanodelivery-based formulations in tumors. Reproduced from ref 24. Available under a CC-BY 4.0 license. Copyright 2023 Shi et al.; c) Benefits of curcumin encapsulation as CLEN. Reproduced from ref 31. Available under a CC-BY 4.0 license. Copyright 2020 Gupta et al.; d) Theranostic application of nanoparticles in cancer therapy and diagnosis of multifunctional carbon-based nanoparticles. Reproduced from ref 26. Copyright 2023 American Chemical Society.

Over time, nanocarrier-based drug delivery systems have taken on multiple distinct configurations, including molecular-level-loaded, surface-loaded, matrix-loaded, and cavity-loaded nanocarriers, as classified by Wang et al.^32^ Our research group recently published a review detailing the compositions and characteristics of these nanocarrier designs, elucidating critical aspects of the process and shedding light on factors that influence their efficacy and biocompatibility.^33^ Additionally, recent papers have provided rich commentary on the growing potential of nanocarriers in healthcare and biomedical research.^34–36^ Despite their revolutionary impact in medicine and diagnostics, nanocarriers still pose key challenges regarding their toxicity, commercial scalability, regulatory oversight, and some design considerations. In this paper, we comprehensively discuss state-of-the-art drug nanocarriers as a snapshot of the last 5 years. We aggregate emergent nanocarrier applications from a translational perspective, focusing on how nanomedicines may further advance medicine. For this, we provide insights into the key nanocarriers presented in contemporary literature, their clinical applications, and the challenges yet to be overcome in nanocarrier development and translational deployment.

## 2 Nanocarrier Systems in Drug Delivery

### 2.1 Polymeric Nanoparticles

Polymeric nanoparticles (Poly-NPs) comprise nanoparticles (NPs) of synthetic biocompatible polymers such as polylactic-*co*-glycolic acid (PLGA), polyethylene glycol (PEG), poly­(vinyl alcohol) (PVA), and polylactic acid (PLA) or naturally occurring polymers such as cellulose, hyaluronic acid (HA), starch, and chitosan. One critical advantage of Poly-NPs is their versatility in drug-loading. They can incorporate a wide range of therapeutic agents, including small molecules, proteins, peptides, and oligonucleotides.^37,38^

Poly-NPs are prepared in a variety of ways that provide control over the physicochemical properties determining drug-loading and release behavior.^39^ Self-assembled Poly-NPs are formed when discrete polymer chains spontaneously order into well-defined nanostructures—a process driven by thermodynamic equilibration and intermolecular forces. In nanoprecipitation, prepolymerized chains self-assemble due to sudden desolvation into well-defined nanostructures determined by the experimental conditions of polymer concentration and solvent chemistry among others. Some other methods have been employed such as ionic gelation, *in situ* polymerization, and self-assembly, as well as atomization or spray drying of polymer emulsions and suspensions. Template-driven assembly of Poly-NPs is another approach that has been more recently explored and offers the advantages of delicate control over the shape and morphology of the NPs, allowing irregularly shaped Poly-NPs and Poly-NPs with hollow cores to be reproducibly synthesized.^40^

Drugs are usually loaded into Poly-NPs by surface adsorption, matrix dispersion, or encapsulation. Depending on their structural organization, they can be further classified into nanocapsules and nanospheres (Figure 1b). In nanocapsules, a polymeric shell surrounds a liquid or semisolid core, whereas nanospheres are solid, matrix-type systems.^41^ Drug-loading in these systems is by passive or active linkage. Passive loading techniques are simple and scalable chemical processes that cause the accumulation of drugs within the NP structures through hydrophobic or electrostatic physisorption. On the other hand, active loading involves chemical linkages rationally designed to reversibly attach drug molecules to functional groups on the Poly-NPs. Active linkage provides precise control over drug release but is comparatively cost-intensive. For example, Miele et al. designed a core–shell Poly-NP to deliver electrostatically loaded anti-HIV RNA-interfering oligonucleotides, overcoming stability and immunogenicity issues of siRNA and alternative delivery modalities such as viral vectors.^42^ Similarly, active linkage of cinnamaldehyde, an antibacterial agent, to a Poly-NP backbone by acid-labile acetal linkages provided pH-sensitive drug release from the construct.^43^

In general, Poly-NPs are a versatile class of nanocarriers with immense clinical potential. Poly-NP surfaces can be functionalized with targeting ligands or stealth polymers such as polyethylene glycol (PEG) to enhance target specificity and prolong circulation time in the body.^44^ This targeted delivery approach can minimize off-target effects, improve the therapeutic index of the encapsulated drugs, and offer the possibility of triggered or stimuli-responsive drug delivery.^45^ Poly-NP-based drug delivery also protects active principles from degradation, increasing their stability and shelf life, which is crucial for maintaining the potency and efficacy of the therapeutic agents.^46^ Poly-NPs have notable advantages over other nanocarrier systems. They offer better stability, controlled release properties, and ease of surface modification than lipid-based nanoparticles.^39^ They are also typically more biocompatible and biodegradable than inorganic nanocarriers such as gold or iron oxide, which may accumulate in the body and cause toxicity concerns.^47^

### 2.2 Lipid-Based Nanoparticles

Lipid-based nanoparticles (LNPs) are a significant advancement in the field of drug delivery, having been developed from cationic and pH-sensitive lipid-coated nucleic acid capsules deployed in the 1980s.^48^ They offer a versatile platform that can be tailored to meet the specific needs of various therapeutic applications.^39,49^ LNPs are characterized by a unique structure, typically consisting of a core surrounded by a shell of amphiphilic molecules such as phospholipids or surfactants that stabilize the core–shell structures. LNPs can solubilize and deliver diverse therapeutic agents including small molecules, peptides, proteins, and nucleic acids.^50^

Based on their structures and components, LNPs are further classified as Liposomes, Solid Lipid Nanoparticles (SLNs), and Nanostructured Lipid Carriers (NLCs).^51,52^ Liposomes are self-assembled vesicles composed of phospholipid bilayers, encapsulating aqueous cores.^48^ In SLNs, solid lipid cores are surrounded by amphiphilic shells.^53^ The drug-loading and long-term stability drawbacks of SLNs prompted the development of NLCs, which essentially incorporate mixtures of solid and liquid lipid molecules in the core matrix to reduce its crystallinity, improve drug-loading, and reduce leakage.^54^ Recently, hybrid drug delivery systems have been developed, combining the beneficial properties of LNPs and other nanocarrier materials such as Poly-NPs. These lipid-polymer hybrid nanocarriers structurally comprise drug cores enclosed in a polymer layer with an outer functionalized lipid coating, providing better mechanical integrity to achieve better stability, reduced drug leakage, and efficient drug entrapment.^55^

The lipid components of LNPs can vary widely and, as presented in Figure 2c,d, comprise physiological lipids such as triglycerides, fatty acids, and cholesterol, which are generally biocompatible and biodegradable, reducing the risk of toxicity associated with certain other nanoparticle systems. The scalability of LNP production has been crucial for their commercial viability and widespread application in drug delivery. LNPs can be manufactured using both solvent-based and nonsolvent-based techniques such as microemulsion and high-pressure homogenization (HPH), which are simpler and more cost-effective techniques than those used for polymeric nanoparticles.^48^ Additionally, the versatility of LNPs expands their potential applications across multiple disease areas.

**Figure 2:**
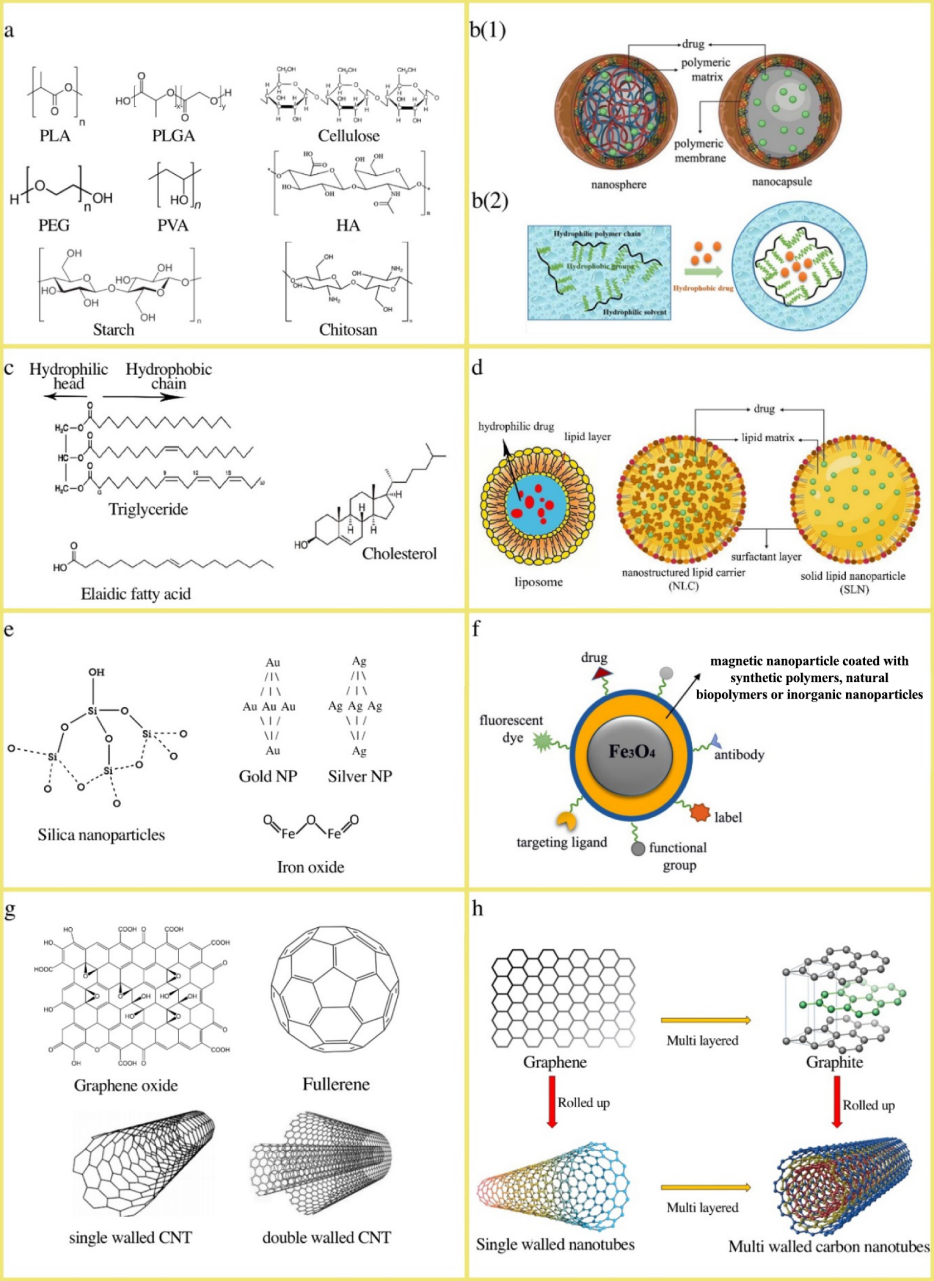
Chemical structures and schematic representations of various nanoparticles utilized in drug delivery systems. a) Synthetic and natural Poly-NPs; b) b(1) Quintessential nanosphere and nanocapsule structures. Reproduced from ref 83. Available under a CC-BY 4.0 license. Copyright 2020 Baldim et al. b(2) A hydrophobic drug encapsulated within an amphiphilic Poly-Np to stabilize it in a hydrophilic solvent environment; c) Some lipids used to prepare LNPs; d) Types of LNPs. Adapted from ref 83. Available under a CC-BY 4.0 license. Copyright 2020 Baldim et al.; e) Inorganic nanoparticles; f) Possible modifications of a magnetic nanocarrier; g) Carbon-based nanoparticles; h) Structural features of key carbon-based nanocarriers. Reproduced from ref 26. Copyright 2023 American Chemical Society.

Within LNPs, SLNs and NLCs exhibit significant advantages over liposomes. Compared to liposomes, for instance, SLNs and NLCs are more physically stable and have therefore garnered considerable attention and widespread production.^56,57^ Additionally, while liposomes and SLNs are more susceptible to degradation and leakage, NLCs have been designed to enhance the nanocarrier’s stability, thereby improving shelf life.^48,58,59^ Commercially, LNPs recently caught global attention, having been the nanocarriers of choice for COVID-19 mRNA vaccine delivery.^60,61^ They have also been formulated severally to deliver highly unstable nucleic acid therapeutics and poorly soluble drugs.^48,61^

### 2.3 Inorganic Nanoparticles

Inorganic NPs are a diverse class of nanocarriers, comprising metals, metal oxides, silica, and other inorganic nanomaterials, which confer unique optical, magnetic, and thermal properties that make them attractive for drug delivery applications.^62,63^ Inorganic NPs generally share exceptional material properties that make them versatile drug nanocarriers. For example, the interesting plasmonic properties of gold NPs have been exploited for photothermal therapy and phototriggered drug release.^64,65^ Similarly, iron oxide NPs have been incorporated into nanocarrier systems for magnetic targeting and imaging functions due to their superior magnetic properties.^66^ As exemplified by plasmonic gold NPs and paramagnetic iron oxide NPs, inorganic nanocarriers have properties that can be easily engineered to design multifunctional platforms with theranostic capabilities.

The structural composition of inorganic nanoparticles is often defined by a solid core or hollow inorganic nanomaterial, surface-functionalized to enhance biocompatibility, targeted delivery, and drug-loading efficiency. Luther and colleagues described payload thiol conjugation to a monolayer-coated gold core, providing improved cellular uptake and cell-specific targeting.^62^ Similarly, thermally stable and chemically inert mesoporous silica NPs (MSNs) are synthesized using surfactant-stabilized micellar templates, resulting in honeycomb-structured constructs with hollow core channels where drugs can be physically or covalently loaded.^62^ On some occasions, inorganic NP-based nanocarriers have been coated with polymeric molecules to improve their colloidal properties and biocompatibility and provide more avenues for surface functionalization. This has, for example, manifested in a chitosan-coated multilayered iron-oxide and gold nanocomposite for doxorubicin delivery.^67^ Additionally, polymeric coatings are often incorporated into inorganic nanocarriers to prevent oxidation of the inorganic nanoparticles and as steric barriers to prevent agglomeration, opsonization, and the residual magnetization associated with magnetic nanocarriers.^67–69^ Metal–organic frameworks (MOFs) have also been employed in conjunction with organic hydrogels to achieve highly porous structures that can load active targeting ligands and drugs and leverage the magnetic properties of the MOFs for multimodal targeting and specific drug delivery.^70^

Inorganic particles are advantageous in their well-defined physicochemical properties and ease of engineering. They also have relatively higher surface area-to-volume ratios that give them better drug-loading efficiency profiles, coupled with their interesting thermal, magnetic, electronic, and optical properties that are applicable in theranostic and targeted delivery systems. However, inorganic nanoparticles, particularly those containing heavy metals, have potential toxicity concerns. The long-term biocompatibility and clearance of inorganic nanoparticles remain areas of active research. Complexities in their synthesis and functionalization can also pose hurdles in scaling up production, making it imperative to develop cost-effective and reproducible manufacturing workflows.

### 2.4 Carbon-Based Nanoparticles

Carbon-based NPs are some of the most thoroughly researched nanomaterials. They have found use in energy applications, electronics, packaging, purification, and several other industries. Carbon-based NPs generally present high surface area-to-volume ratios and tunable nanoscale morphology and surface chemistry, making them suitable as nanocarriers with enhanced entrapment efficiency and ligand-functionalized targeted delivery capabilities. Structural forms of carbon-based NPs that have been developed in the literature include carbon nanotubes, graphene oxide, fullerenes, nanodiamonds, carbon dots, and carbon quantum dots, each of which offers distinct characteristics that make them suitable for specific applications in drug delivery (Figure 2g,h).^29,71–73^

Carbon nanotubes (CNTs) are one-dimensional or three-dimensional (in the case of multiwalled CNTs) nanostructures that are explored for their high surface area and physisorptive and chemisorptive cargo-loading capacity. Their ability to traverse cell membranes also makes CNTs suitable for intracellular drug delivery, an invaluable pharmacotherapeutic phenomenon in immuno-oncology. The nanocarrier properties and capacities of CNTs are determined by their structural and dimensional features. Molecular dynamics simulations have, for example, reported the dependence of CNTs’ doxorubicin entrapment efficiency on the diameter and chirality of the CNTs, as well as the presence and nature of defects within the nanotubes.^74^

Graphene is a two-dimensional nanomaterial consisting of sp^2^-hybridized carbon atoms arranged in a honeycomb lattice structure with delocalized electrons. The chemical features of graphene endow it with interesting photothermal and electronic properties that have driven its adoption into a wide range of applications. Having a large surface area and π-orbitals of delocalized electrons, the graphene molecule provides ample area for adsorption, through π–π stacking, of aromatic compounds. This has been explored to functionalize graphene as a multidrug carrier.^75^ Some derivatives of graphene such as graphene oxide (GO) and reduced graphene oxide (rGO) have also been developed for the delivery of antineoplastic agents, anticoagulants, and nucleic acid payloads, among others.^75^ Other carbon-based nanocarriers have also been used in drug delivery. Carbon dots, for example, are spherical carbon-based NPs, less than 10 nm in size and rich in carboxyl, hydroxyl, and amino functional groups, that have found recent success in drug delivery to the central nervous system.^76^

The structural features of carbon-based NPs determine their pharmacokinetic properties as drug carriers. The needlelike structure of CNTs, for example, allows them to penetrate cell membranes more efficiently than spherical carbon-based NPs.^77^ Carbon-based NPs also frequently have reactive side groups that can be functionalized for targeted delivery and improved biocompatibility. Their chemical properties render them amenable to covalent and noncovalent functionalizations that serve diverse purposes of therapeutic values.^77,78^ These have resulted in targeting ligand-functionalized carbon NP-based drug delivery systems as well as theranostic platforms with real-time drug delivery and response monitoring.^26,79^ Like inorganic NPs, certain carbon NPs such as graphene and CNTs have unique optical and electronic properties that have been directly exploited in the literature for photothermal therapy, bioimaging, and actuated drug release.^80,81^ CNTs exhibit strong optical absorbance in the near-infrared region, converting photonic energy to heat—a physical phenomenon underlying their use in photothermal therapy and light-controlled drug delivery. Combining multiwalled CNTs with plasmonic gold NPs has achieved synergistically high efficacy in PTT against breast cancer cells.^82^

Despite the merits of carbon-based NPs as nanocarriers, they still face peculiar challenges in healthcare, stemming from their potential toxicity concerns, limited biodegradability, clearance, and synthesis reproducibility. Carbon-based NPs require precise control over process parameters during synthesis to control the size, purity, and functionalization. This also translates to higher production costs and complexity, constituting a drawback for large-scale production.^73^ Despite these challenges, carbon-based NPs offer unique advantages for drug delivery due to their high drug-loading capacity, improved cellular uptake, versatile functionalization, and multifunctional capabilities. Nonetheless, intelligent nanoparticle design, surface modification, and thorough characterization approaches are required to ensure their safe and effective use as nanocarriers.

The chemical and structural features of some Poly-NPs, LNPs, inorganic NPs, and carbon-based NPs that have been adopted as nanocarriers are illustrated in Figure 2.

## 3 Potential Clinical Applications of Nanoparticle-Based Drug Delivery Systems

### 3.1 Cancer Therapy

Current clinical approaches to managing cancers involve chemotherapy, immunotherapy, radiotherapy, and surgical procedures. While these have largely resulted in significant strides over the past few decades, research is ongoing to improve oncologic pharmacotherapies, enhancing their efficacy and toxicity profiles through optimization of pharmacokinetic parameters and targeted, controlled *in vivo* delivery. Accordingly, nanocarriers have been explored due to their intrinsic properties that can overcome challenges associated with antineoplastic APIs, such as poor solubility, nonspecific biodistribution, and adverse effects. Some of the nanocarriers adopted for cancer therapy include Poly-NPs, liposomes, and inorganic NPs, which have been investigated to deliver both single and combination therapies.^33^

The unique properties of nanocarriers can be exploited to enable passive preferential accumulation and cargo release at tumor sites associated with leaky vasculature and impaired lymphatic drainage, a phenomenon typically referred to as Enhanced Permeability and Retention (EPR). As discussed earlier, nanocarriers can also be functionalized for active targeting strategies to augment EPR through the conjugation of target-specific ligands.^84,85^ There are several instances of these approaches to enhancing cancer therapy in contemporary literature.

Crucially, smart nanocarrier systems have emerged as one such nanomaterial-based approach to antineoplastic drug delivery. Smart nanocarriers leverage established biophysical principles and well-researched material properties to achieve predictable and controllable delivery phenomena. They offer promising advancements over conventional cancer therapy, enabling a more sophisticated treatment strategy. Smart nanocarrier systems often consist of a core nanomaterial with a peculiarly desirable property functionalized with a combination of targeting ligands, biopolymer coatings, and/or actuator molecules for stimulus or signal responsivity. Gold NPs are commonly used in the core of these systems due to the ease of their synthesis, particle size and morphology control, ease of surface functionalization, and biocompatibility stemming from their relative chemical inertness. Nonetheless, some other inorganic NPs, Poly-NPs, and carbon NPs have been adopted singly and in combinations.

Xu et al. developed a porous gold nanoshell construct, loaded with emtansine together with a photosensitizer for synergistic chemotherapy upon near-infrared irradiation, achieving on-demand drug delivery, remarkable tumor regression, and prolonged survival in *in vivo* models.^86^ Similarly, Hou et al. designed a smart nanocarrier system comprising a nanoporous silica core functionalized with plasmonic silver quantum dots and hyaluronic acid for the multistimulus-responsive delivery of doxorubicin.^87^ Dai et al. also reported the development of a theranostic nanocarrier system for simultaneous imaging and photothermal therapy.^88^ This system, composed of a gold nanorod core and an MSN shell, could efficiently accumulate in tumors and generate heat upon near-infrared light irradiation (NII), leading to tumor ablation, while facilitating NII-mediated delivery of a small molecule inhibitor of the immune checkpoint protein, PDL1, as well as a vaccine stimulating *in vivo* production of an anti-VEGF antibody. Other studies have designed multifunctional smart nanocarrier systems that respond to pathophysiological cues such as pH, enzymatic activity, and redox reactions, as well as external stimuli such as photoradiation, sound, electric, and magnetic fields.^4^

In contrast to smart nanocarrier systems, much simpler nanocarrier systems have also been designed, exploiting simple material properties for direct cytotoxicity and EPR, wherein the NP adopted itself demonstrates the desired antineoplastic and/or targeting effect. Designing one such system, Abdellatif et al. investigated chitosan-capped silver nanoparticles with inherent activity against breast cancer cells.^89^ The schematic illustration of this simple design is presented in Figure 3b. Additionally, several studies have reported the *in vitro* anticancer activities of selenium nanoparticles against breast, lung, colorectal, prostate, cervical, and liver cancer cell lines, mediated by their inhibition of cancer metastases, paving the way for subsequent studies and optimizing the synthesis and morphology of these simple nanomaterial systems for potential antineoplastic applications.^90^

**Figure 3:**
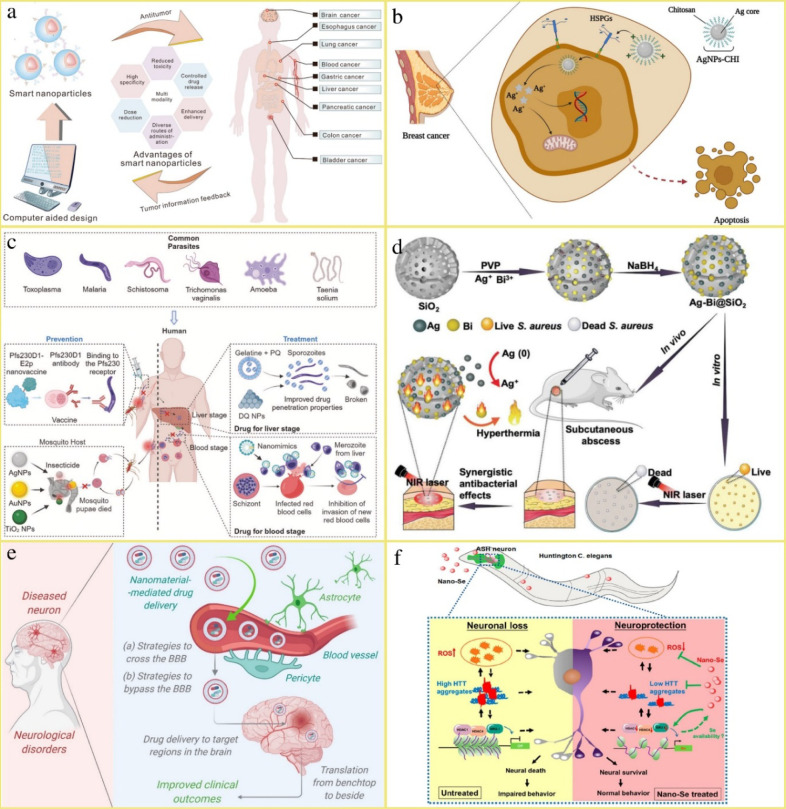
Illustrations of a) Smart nanoparticles’ multifunctional use in cancer management. Reproduced from ref 4. Available under a CC-BY 4.0 license. Copyright 2023 Sun et al.; b) The proposed mechanism of the selective anticancer activity of Ag NPs-CHI against breast cancer cells. Reproduced from ref 89. Available under a CC-BY 4.0 license. Copyright 2023 Abdellatif et al.; c) Antiparasitic applications of nanocarriers. Reproduced from ref 118. Available under a CC-BY 4.0 license. Copyright 2024 Huang et al.; d) Preparation of Ag–Bi@MSNs and its synergistic antibacterial effects. Reproduced with permission from ref 112. Copyright 2020 WILEY-VCH Verlag GmbH & Co.; e) Nanocarriers’ capacity to facilitate CNS drug delivery by crossing or bypassing the BBB. Reproduced from ref 147. Available under a CC-BY 4.0 license. Copyright 2021 Faiyaz et al.; f) Nano-Se as an efficient approach to treating HD demonstrated in a model. Reproduced from ref 178. Copyright 2019 American Chemical Society.

Nanocarriers have recorded tremendous translational success in cancer, and several nanocarrier-based formulations have already received clinical approval. These include Doxil (liposomal doxorubicin), Abraxane (albumin-bound paclitaxel), and Onivyde (liposomal irinotecan).^84,85,91^ An overview of contemporary studies that have examined nanocarriers for the delivery of anticancer agents is presented in Table 1.

**Table 1:** Nanocarriers Developed for Anticancer Delivery

	Nanocarrier	Functionalization/Modification	API	Disease/Cell Lines	Studies	ref
1	Manganese NPs	Platelet membrane coating	Indocyanin Green and amidated indoximod	4T1 mammary tumor cell line	*In vitro* photothermal and photodynamic effect; *in vitro catalytic* oxidation; *In vitro* cytotoxicity assay; *In vivo* tumor inhibitory study, toxicity, and immune activation studies.	^92^
2	Chitosan and Polypyrrole Co-Poly-NP	TiO_2_ surface adsorption	Oxaliplatin	Colorectal cancer	Characterization; *in vitro* ROS generation; drug-loading and ultrasound-responsive release; *in vitro* cytotoxicity; *in vivo* anticancer efficacy, safety, and immune activation.	^93^
3	Gold	Gelatin Biopolymer coating	Methotrexate	Human Breast Cancer (MCF7)	Drug-loading; entrapment efficiency; drug release; *in vitro* MTT assay.	^94^
4	Iron Oxide – Silica NP core	Cancer Stem Cell coating	P38 inhibitor	Stress-escaping tumor cells	Characterization; *in vitro* cellular uptake and cytotoxicity; *in vivo* tumor imaging, anticancer activity.	^95^
5	Gold NP	PSP001 polysaccharide coating	Doxorubicin and anti-HER siRNA	Breast cancer	Characterization; drug release; *in vitro* serum stability and hemo-compatibility; *in vitro* cytotoxicity and gene-silencing; *in vivo* xenograft breast cancer model, anticancer effects, biodistribution, and gene knockdown effects.	^96^
6	Cationic Gelatin	-	Paclitaxel	Lung Cancer (A549), Colon Cancer (HT29)	Drug-loading and Release; MTT cell viability assay.	^97^
7	Niosomal Nanoparticle	Hyaluronic Acid	Epirubicin	Mammary tumors	Entrapment Efficiency; Drug release; *In vitro* cytotoxicity; cellular uptake; *In vivo* histopathology studies.	^98^
8	Aluminum Nitride Nanotubes	5-ASA surface adsorption	5 – Acetyl Salicylic Acid	Colorectal Cancer	Adsorption studies via density functional theory	^99^
9	MSN	Lactobionic acid-modified carboxymethyl chitosan surface coating	Curcumin	Hepatocellular carcinoma	Characterization; drug release kinetics; *in vitro* cytotoxicity and wound healing; *in vivo* antitumor and immune activation studies.	^100^
10	Graphene Quantum Dots	Magnetic Nanocomposites, Folic Acid	Curcumin	Breast Cancer (MCF-7); Osteosarcoma (MG-63)	Physicochemical characterization; drug release; *In vitro* MTT assay.	^101^
11	Silver Nanoparticles	Chitosan coating	-	Human Breast Cancer Cells	Material characterization; shelf life and stability; *In vitro* cytotoxicity assay; ELISA against cancer biomarkers.	^89^
12	Gold Nanorod-Mesoporous Silica core–shell nanostructure	-	BMS1166 and an Anti-VEGF peptide vaccine	Hepatocellular carcinoma	Characterization; photothermal analysis; biodistribution *In vitro* cytotoxicity assay; *in vivo* animal cytotoxicity study; *In vivo* photothermal immunotherapy.	^88^
13	MSNs	Cancer cell membrane coating	Doxorubicin and miR-34a	Triple-negative breast cancer	Drug-loading and release; cellular uptake; *in vitro* cytotoxicity; *in vivo* biotoxicity and antitumor efficacy.	^102^
14	Porous Gold Nanoshell	Methoxy-PEG, trastuzumab	A Maytansine Derivative	Breast Cancer	Characterization; Photothermal conversion; redox-mediated drug release; photoacoustic and photothermal imaging; *In vitro* cytotoxicity; *In vivo* cellular uptake; biodistribution and cytotoxicity.	^86^
15	Chitosan-coated PLGA NPs	Functionalized with targeting aptamer and folate	Quercetin and Sorafenib	Breast Cancer cells (MCF-7 and MDA-MB-231)	Characterization; drug release; *in vitro* cytotoxicity assays; cellular uptake assays.	^103^
16	Dendrimer-encapsulating Gold Nanoparticles	Hyaluronic Acid	Doxorubicin	Ovarian Cancer	Drug-loading; chemical stability; pH-responsive drug release; *In vivo* tumor xenograft study.	^104^
17	Polymeric micelles	-	A4 – a DNA repair inhibitor	Colorectal Cancer	Copolymers characterization; drug-loading efficiency; *In vitro* release; cytotoxicity assay; assessment of synergy with carboplatin and oxaliplatin.	^105^
18	DSPE – PEG_2000_ Liposome	EGFR-targeting	Irinotecan	Colorectal Cancer	Characterization; *in vitro* anticancer effect; *in vivo* tumor xenograft model.	^106^
19	Porous Silica Nanocarrier	Hyaluronic Acid; Silver sulfide quantum Dots	Doxorubicin		Characterization; CD44 receptor targeting; photothermal therapy potential.	^87^
20	Graphene Oxide		Doxorubicin		Molecular dynamics simulation	^107^

### 3.2 Infectious Diseases

Infectious diseases are often difficult to treat radically due to conventional treatment challenges such as antimicrobial resistance, which develops due to subtherapeutic microbial site accumulation. Infectious diseases, caused by a wide array of pathogenic microorganisms, are typically more prevalent in the developing world, where logistical challenges compound disease treatment with supply chain inefficiencies, drug stability issues, and healthcare financing models that result in low patient adherence, coupled with drug-related toxicities and extended therapeutic regimens. To address these challenges and shore up antimicrobial therapy options, especially considering recent global and regional epidemics, several approaches have been explored on both scientific and policy framework fronts.^108^ Nanomaterials, with their potential for advancing diagnosis and treatment of infectious diseases, have emerged as one of the forerunners of technologies to combat infectious diseases in contemporary times.

Nanocarriers have shown promise in formulations delivering different antimicrobial classes, including antimicrobial peptides, vaccines, oligonucleotide molecules, and small-molecule antimicrobial agents against several pathogens of clinical significance. The value of nanocarriers in these formulations arises from their improvement of the active principles’ solubility, stability during storage, and persistence within the physiological environment, as well as the possibility of functionalizing these systems for passive or active targeting and accumulation at target sites. Some NPs also possess antimicrobial activities themselves. These properties, along with smart, stimulus-responsive delivery principles, improve therapeutic outcomes by overcoming common mechanisms through which pathogens develop and exert antimicrobial resistance.^109^ Additionally, the controlled release of active principles from nanocarrier systems circumvents the need for frequent dosing, improving patient adherence, while maintaining therapeutic serum levels and target site accumulation of the antimicrobial agents, leading to an overall enhancement of clinical and humanistic outcomes of infectious disease therapies.

Many instances of anti-infectious agents formulated in nanocarrier systems have been reported in recent literature. These include Poly-NPs explored for antiretroviral delivery in HIV/AIDS and inorganic NPs, such as silver and gold, investigated for their antimicrobial properties and potential applications in wound healing and infection control, among other such applications.^110,111^ In a study conducted by Cao et al., MSN-supported silver–bismuth nanoparticles (Ag–Bi@SiO2 NPs) were developed for enhanced antibacterial treatment, combining hyperthermia and the antimicrobial activity of silver against methicillin-resistant (MRSA) (Figure 3d).^112^

The Pfizer-BioNTech and Moderna COVID-19 vaccines utilized LNPs to encapsulate and deliver mRNA encoding the SARS-CoV-2 spike protein, eliciting a robust immune response. Building on the success of LNP-delivered mRNA vaccines, a more recent study by the COVARNA consortium developed multiple LNP- and Poly-NP-based nanoemulsion and nanocapsule prototypes in delivering mRNA vaccines against SARS-CoV-2.^113^ In addition to antiviral agents and vaccines, nanocarrier systems have also been developed against other difficult-to-treat infectious diseases such as mycobacteria, characterized by multidrug resistance, polypharmacy, and long treatment periods. SLNs, loaded with the antibiotic rifampicin and surface-functionalized with mannose for targeted delivery to , exhibited enhanced intracellular uptake and improved efficacy against drug-resistant strains, offering a promising approach for combating antimicrobial resistance in mycobacteria.^114^

Antimicrobial peptides and other immunotherapeutic antimicrobials are some other classes of anti-infectives that have been formulated in nanocarrier systems. Zhang et al. reported the development of gold nanoparticles coated with ultrashort antimicrobial dipeptides for treating bacterial infections. The nanoconstruct demonstrated potent antibacterial activity against multidrug-resistant strains, including MRSA.^115^ In a similar study, NPs carrying bacterial outer membrane vesicles (OMVs) stimulated immune reactions against .^116^

Nanocarriers and NPs also offer advantages in combating parasitic pathogens. Different self-assembled protein NPs have been reported to target distinct stages of the malaria parasite’s lifecycle, for example.^117^ This has given rise to nanovaccines designed to generate antibodies against plasmodial species while also possessing the size and mobility to traverse the lymphatic system presenting antigenic material on MHC molecules to elicit a sustained cellular immune response.^117,118^

Leveraging the unique properties of NPs, innovative strategies to combat infectious diseases are being developed, offering improved therapeutic outcomes and addressing challenges such as drug resistance and targeted delivery. The FDA has approved several NP-based formulations for the treatment of infectious diseases, including AmBisome (liposomal amphotericin B) for invasive fungal infections and Abelcet (lipid complex amphotericin B) for severe fungal infections in patients intolerant to conventional amphotericin B.^4^ Additionally, intricate designs of nanocarriers have been exploited for multifunctional purposes, exemplified by a recent nanogel designed for antimicrobial and enamel remineralization purposes,^119^ further demonstrating the translational potential of nanocarrier systems. Some of the important recent advances in nanocarrier-based anti-infective formulations are summarized in Table 2.

**Table 2:** Nanocarriers Developed for Antimicrobial Drug Delivery

	Carrier	Functionalization/Modification	API	Disease	Studies	ref
1	Nanoemulsions, Nanocapsules, Lipid Nanoparticles	PEGylation (of LNP)	mRNA coding for Receptor-Binding Domain	SARS-CoV-2	*In vitro* toxicity and bioactivity; *in vivo* animal studies	^113^
2	SLNs	Mannose surface modification	Rifampicin	Tuberculosis	Characterization; *in vitro* macrophage cytotoxicity (MTT assay); *in vitro* antibacterial and antibiofilm assay.	^114^
3	Gold	Antimicrobial peptide surface adhesion	Ultrashort antimicrobial dipeptides	Multidrug-resistant bacteria	Characterization; *in vitro* antibacterial assay; hemolysis and cytotoxicity assay; molecular dynamics simulation; metabolic study and acute toxicity; *in vivo* antimicrobial assay;	^115^
4	Copper NPs	Hyaluronic acid grafting	Luteolin	Bacterial prostatitis	Characterization; drug release; *in vitro* antimicrobial activity and biocompatibility; *in vivo* prostatitis efficacy, histopathology, and biocompatibility studies.	^120^
5	Self-Assembled Nanoprotein	-	FMP014 – containing multiple falciparum CD4 and CD8 epitopes	Malaria	Undergoing Clinical Trials	^117^
6	Self-organized Nanoprotein	Fusion to IMX313; a hybrid protein to improve biocompatibility	Pfs25 – a malaria transmission antigen	Malaria	Preclinical evaluations and Clinical Trials	^121^
7	Outer Membrane Vesicles	-	Immunogenic vesicles produced by mutant bacterial strains	Shigellosis	*In vivo* immunization studies; *in vitro* adhesion/invasion assay; agar plate antibacterial assay.	^116^
8	SLNs	Mannose coating	Isoniazid		Cellular uptake; pH-sensitive drug release; intracellular antibiotic efficacy; *in vivo* antibiotic efficacy assay.	^122^
9	Liposomes	Surface functionalization with targeting anti-CD4 antibody and peptide dendrimer	Dolutegravir and Lamivudine	HIV/AIDS	*In silico* evaluation; morphology and characterization; entrapment efficiency; *in vitro* release; cellular uptake and cytotoxicity.	^123^
10	Liposome	-	Olive leaves polyphenols	MRSA	Characterization; *in vitro* stability; encapsulation efficiency; *in vitro* release and antimicrobial studies – MIC and MBS estimation.	^124^
11	Liposome-Polymer Hybrid NPs	-	A Multi-Epitope DNA Vaccine	Multiple Infectious Diseases	Structural and morphological characterization; encapsulation efficiency; *in vitro* DNA release; cytotoxicity; transfection efficiency; *in vivo* immunogenicity studies.	^125^
12	Trimethylated chitosan NPs	-	SARS-CoV-2 Spike Protein	COVID-19	Characterization; *In vitro* formulation release; *in vivo* toxicity studies; *in vivo* immunogenicity	^126^
13	Carbon Nanotubes	-	Isoniazid and Fluoxetin	Tuberculosis	Characterization; *In vitro* antimicrobial assays; gene expression and cytokine quantification assays.	^127^
14	Chitosan-Cyclodextrin hybrid NPs	HA and Tyrosine functionalizations	Baicalin		Characterization; encapsulation efficiency; *In vitro* MIC quantification; *In vitro* and *In vivo* biofilm elimination studies.	^128^
15	SLN and Chitosan NPs	-	Cinnamon Oil	Multidrug-Resistant and	Characterization; *In vitro* antimicrobial assays; encapsulation efficiency; drug release; biocompatibility assay	^129^
16	SLN and Chitosan	-	Antibacterial phytochemical;	and	Physicochemical evaluation; *In vitro* drug release; *In vitro* antibacterial assay; biocompatibility assay.	^130^
17	Zinc Oxide NPs	-	and nitazoxanide	Cryptosporidiosis	Morphological characterization; zeta potential; *In vivo* efficacy studies.	^131^
18	Niosomes	Aptamer surface modification	Propolis	Tuberculosis	Characterization; Drug entrapment efficiency; *In vitro* release profile; Mycobacterial-targeted distribution; *In vitro* biocompatibility assay; antimycobacterial activity.	^132^
19	Hyaluronic Acid-wrapped NP	-	Curcumin-Copper complex	Bacterial Prostatitis	Characterization; *In vitro* antibacterial activity; cytocompatibility; anti-inflammatory assay; *In vivo* antiprostatitis studies; *In vivo* biocompatibility and organ toxicity.	^133^
20	Trimethylated chitosan NPs	-	SARS-CoV-2 Spike Protein	COVID-19	Characterization; cellular uptake; *In vivo* mouse immunogenicity studies.	^134^
21	Poly-NPs (Chitosan, Gellan gum, and dextran)		Nisin		Characterization; drug-loading; *In vitro* drug release; disk-diffusion antimicrobial assay; *In vitro* cytotoxicity (MTT assay).	^135^
22	Carboxymethyl Chitosan/Lysozyme Nanogel	-	Amorphous Calcium Phosphate and Antimicrobial agents		Characterization; antimicrobial assay; *in vitro* mineralization assay; *in vivo* animal assays.	^119^

### 3.3 Neurodegenerative Disorders

Neurodegenerative disorders are chronic diseases, often requiring lifelong management with multiple drugs and complex regimens that mostly only provide symptomatic relief. The management of neurodegenerative disorders is often further complicated by biodistribution challenges encountered due to the physiological role of the blood–brain barrier (BBB). The physicochemical properties of most APIs used in managing neurodegenerative disorders render them inefficient in crossing the BBB. Further, bioimaging, diagnostic, and disease monitoring strategies are hampered by the impaired distribution of biosensing constructs into the central nervous system. In multiple recent studies, nanocarrier systems have shown promise in addressing these and other challenges peculiarly associated with managing neurodegenerative disorders, such as Alzheimer’s disease (AD),^136–138^ Parkinson’s disease (PD),^17,139,140^ and multiple sclerosis.^141–143^

NPs can afford more efficient transport through the BBB, leveraging one or more researched mechanisms such as adsorptive-mediated transcytosis, receptor-mediated transcytosis, and cell-mediated transport.^144,145^ Accordingly, nanocarrier systems have been reported on multiple occasions to effectively deliver cargo to the central nervous system, offering a competitive advantage over conventional formulations for the same purpose. This is especially important in the context of smart nanocarrier systems with which both normal healthy features and pathophysiological changes in the BBB can be targeted to deliver cargo only in intended *in vivo* conditions. In a recent study, Nong et al. conjugated LNCs with antibodies that bind cell adhesion molecules (VCAM) expressed at the BBB to enable targeted delivery to the inflamed BBB in acute ischemic stroke. Anti-inflammatory drugs administered intravenously after ischemic stroke reduced cerebral infarct volume by 62% (interleukin-10 mRNA) or 35% (dexamethasone) only when they were encapsulated in VCAM-targeted LNCs.^146^

Nanocarrier constructs can either cross the blood–brain barrier (BBB) effectively or bypass it altogether to reach specific central nervous system regions. This additional unique ability afforded by the physiochemical profile of NPs provides immense clinical benefits (Figure 3e). Their importance is highlighted in their continued use for formulating numerous neurotherapeutic agents ranging from small molecules to phytochemicals and peptides, among others.^147^ In a recent study, chitosan Poly-NPs achieved direct nose-to-brain delivery of donepezil hydrochloride, bypassing the BBB, to treat AD.^148^ Confocal micrography studies confirmed delivery to the brain by the LNPs, which delivered the API almost thrice as well as intranasal and 10 times more than oral donepezil formulations. A similar study explored intranasally administered magnetic nanoparticles for bioimaging of target brain regions, potentially leveraging the BBB-bypassing capability of the nanocarrier.^149^

Supplementation of essential molecules that are deficient in neurodegenerative diseases has also been improved with nanocarrier systems. Cong et al. explored selenium nanoparticles (Nano-Se) for Huntington’s disease (HD) therapy in transgenic () models. At low doses, Nano-Se significantly decreased neuronal death, improved behavioral function, and protected against damage caused by stress (Figure 3f). An overview of nanocarrier-based systems developed for neurodegenerative disease management is presented in Table 3.

**Table 3:** Nanocarriers Developed for Managing Neurodegenerative Diseases

	Carrier	Functionalization/Modification	API	Disease	Studies	ref
1	Chitosan		Donepezil HCL	AD	Characterization; *in vitro* drug release; *ex vivo* permeation; *in vivo* pharmacokinetics study; confocal microscopy-based drug localization study.	^148^
2	LNCs	VCAM-targeting antibodies	Anti-inflammatory drugs: Dexamethasone, IL10, mRNA	Ischemic stroke	Transient middle cerebral artery occlusion mouse model of targeted delivery and anti-inflammatory activity on cerebral infarct volume.	^146^
3	Selenium NPs	BBB transport peptide	Resveratrol	AD	*In vitro* stability, plaque aggregation study; BBB transport, cytotoxicity, antioxidation study; *in vivo* AlCl_3_ and D-gal induced AD mice model.	^150^
4	GO Nanosheets	PEG and Polyethylenimine functionalizations	GSK3β knockdown siRNA	AD	Characterization; loading capacity; cellular uptake; cell cytotoxicity; streptozocin-induced *in vitro* and *in vivo* AD models.	^151^
5	Poly-NP and NLP	PEGylation	Entacapone	PD	Characterization; encapsulation efficiency; *in vitro* drug release and stability studies; *in vitro* cytotoxicity; cellular uptake.	^152^
6	Cerium Oxide nanocrystals	Red Blood Cell membranes	Carbon Quantum Dots	AD	Characterization; photothermal conversion and stability; *in vitro* antioxidation study, Aβ inhibition and disaggregation, cytotoxicity, and cellular uptake; *in vivo APP/PS1* transgenic mice model.	^153^
7	PLGA NPs	Polysorbate 80 coating	Thymoquinone	AD	*In vitro* enzymatic assay; *in vivo* AD mouse behavioral model; *ex vivo* brain hippocampal tissue histopathology.	^154^
8	Liposomes	Chemokine receptor type-4 surface modification	Osthole	AD	*In vitro* intracellular distribution; *in vitro* and *in vivo* neuroprotective studies.	^155^
9	MSNs	Amine Modification and polydopamine coating	A GSK3β inhibitor	Traumatic Brain Injury	Characterization; *in vitro* drug-loading and release, cytotoxicity, and antioxidation studies; *in vivo* cryogenic brain injury model.	^156^
10	Human Serum Albumin NPs	-	Melatonin	PD	Characterization; encapsulation efficiency and drug release; *in vitro* and *in vivo* neurotherapeutic efficacy; *in vivo* biodistribution.	^157^
11	Liposomes	Mannose surface functionalization; cell penetrating peptide (CPP)	ApoE	AD	Cytocompatibility study; *in vitro* targeted cargo delivery; *in vivo* efficacy and toxicity studies.	^158^
12	Cobalt-doped Iron Oxide Nanozymes	-	-	Acute Ischemic Stroke	*In vitro* antioxidation and anti-inflammatory studies; *in vivo* focal and prothrombic stroke models.	^159^
13	Catechin Polyphenolic NPs	PEGylation	-	Intracerebral Hemorrhagic Stroke	Characterization; *in vitro* and *in vivo* toxicity studies; intracerebral hemorrhage model in mice.	^160^
14	Poly-NPs	Ce6 conjugation for NIR imaging and poly(PDA-*co*-HEMA) co polymerization for pH responsivity	Rapamycin	Acute Ischemic Stroke	Characterization; *in vitro* drug release, pH responsivity, and cellular uptake studies; *in vivo* neuroprotective studies in transient middle cerebral artery occlusion mouse model.	^161^
15	Platelet membrane vesicles	-	Oxygen gas	Acute Ischemic Stroke	Characterization; entrapment efficiency; *in vitro* release; platelet aggregation assay; neuronal injury protection assay; imaging studies.	^162^
16	Electro-spun Poly-Caprolactone Nanofibers	Polydopamine modification	Stem cell-derived exosomes	Traumatic Brain Injury	Characterization; *in vitro* release studies, anti-inflammatory, and nerve repair studies; *in vivo* controlled cortical impact mouse model; biocompatibility and toxicity studies.	^163^
17	Liposome	Angiopep-2 peptide functionalization	Resveratrol	Age-related neurodegeneration	Characterization; *in vitro* and *in vivo* biocompatibility assays; cellular uptake; *in vitro* neurotoxicity reversal; *in vitro* BBB penetration; *in vivo* targeting; *in vivo* pharmacological evaluation and behavioral studies.	^164^
18	Hollow Mesoporous Prussian Blue NPs	Red Blood Cell membrane-coating	Curcumin and microRNA	AD	Characterization; drug-loading; *in vitro* release; *in vitro* antioxidant study; cytotoxicity and biocompatibility assays; *in vitro* BBB penetration assays; *in vivo* animal models and behavioral studies	^165^
19	Thermosensitive Liposomes	Phospholipase A2 targeting functionalization	HI-6	Acute brain poisoning	Characterization; *in vivo* detoxification potential; toxicity studies; Central Nervous System targeting.	^166^
20	Black phosphorus nanosheets	PEG modification	Matrine	PD	Characterization; *in vitro* safety and biocompatibility; *in vitro* BBB penetration; cellular uptake and colocalization; *in vitro* neuroprotective studies; *in vivo* photothermal studies; biodistribution.	^167^

The translational potential of nanocarriers is further demonstrated in the number of approved nanomedicines, patents, and clinical trials involving these systems in the management of cancers, infectious diseases, and neurodegenerative disorders.^168^ Sorrentino and colleagues recently discussed and outlined nanocarrier-based formulations that have been approved for cancer therapy.^169^ Similarly, a 2024 review by Melo and colleagues comprehensively discusses some randomized clinical trials of nanomedicines in the management of various cancers,^170^ while another review explored the patent landscape of nanomedicines in cancer.^171^ Similar recent reviews have explored the clinical translations of nanocarriers in the management of infectious diseases^172–174^ and neurodegenerative diseases.^175–177^ Some recent nanocarrier systems designed for the management of cancers, infectious diseases, and neurodegenerative diseases, as reported in the literature, are illustrated in Figure 3.

## 4 Limitations of Nanoparticle-Based Drug Delivery Systems

### 4.1 Toxicity, Biocompatibility, and Delivery Barriers

Nanoparticles interact uniquely with biological systems in ways that might introduce biotoxicity. It is important to fully demystify the biodistribution, clearance, and long-term effects of nanocarrier systems in their various configurations.^179^ Recent studies have reported systemic inflammation arising from NPs in multiple toxicology models. CNTs have, for example, triggered inflammatory responses accompanied by lipid dysregulation-mediated granuloma formation in mice.^180^ An earlier study already demonstrated a significant relationship between the morphology of nanotubes with the severity of toxicity, with higher aspect ratio CNTs having more pronounced adverse effects.^181^ Additionally, several studies have reported inflammatory responses with single-walled carbon nanotubes (SWCNTs).^182^

Inorganic NPs, especially metal oxides, may also cause oxidative stress by generating cytotoxic reactive oxygen species (ROS). Defects and vacancies, often associated with copper oxide, zinc oxide, iron oxides, and other metal oxide NPs, can catalyze ROS production through photochemistry or Fenton reactions, resulting in oxidative damage to lipids, proteins, and nucleic acids in the cells.^14,183^ While these mechanisms confer important functionality on the inorganic NPs, they pose a biotoxicity challenge and have been the target of recent biocompatibility optimization research.

Generally, there is ongoing research to better understand NPs’ interactions with biosystems and develop strategies for mitigating potential toxicity, such as surface modifications, or using alternative materials with improved biocompatibility profiles such as Poly-NPs and LNPs, enabling safe use, handling, and production.^184,185^ The biocompatibility and minimal toxicity inherent in natural and biodegradable synthetic polymers such as chitosan and PLGA, due to their chemical similarity to biomolecules, have thus far driven their adoption in biomedical applications.^186^ Similarly, LNPs bear physicochemical similarities to cellular membranes and are thus generally safe with minimal toxicity.^187^

### 4.2 Scale-Up and Commercial Manufacturing

To ensure the commercial viability of nanoparticle-based drug delivery systems, scalable and cost-effective manufacturing processes are being developed. These range from microfluidic technologies to continuous manufacturing processes such as twin-screw extrusion, membrane emulsification techniques coupled with microfluidic devices, and high-pressure homogenization. Other approaches include continuous flow reactors and microreactors, which have been used for the industrial production of protein NPs such as albumin-bound paclitaxel (Abraxane). Anderluzzi et al. demonstrated a scalable manufacturing process for SLNs, optimizing a microfluidizer-based high-shear mixing process followed by a tangential flow filtration workflow, controlling process parameters to achieve desired NP size and polydispersity.^188^ A similar scale-up attempt operationalized two multiinlet vortex mixers for the sequential flash nanoprecipitation of MSNs.^189^ The MSNs loaded with the nematocidal agent, abamectin, achieved high encapsulation rates, maintained nematocidal activity, and had tunable morphology with the optimization of process parameters.

Several other scalable production workflows for NPs based on nanoprecipitation, supercritical fluid technology, extrusion, and microfluidization, among other techniques, have been discussed in recent literature.^190–192^ Crucially, each technique has its peculiar advantages and limitations, necessitating further research to enable scalable, cost-effective, sustainable, and reproducible production of nanocarriers for industrial applicability. It is important to note that there is a lack of comprehensive standards in the characterization and reporting of nanocarriers, resulting in fragmented and frequently uncompilable protocols and findings. This needs to be surmounted with transparency and standardization if nanocarriers are to be adopted at scale and manufactured industrially as they are essential for commercial viability and clinical translation.

### 4.3 Regulatory Challenges

Oversight of the major global pharmaceutical markets is conducted by the FDA in the United States and the EMA in Europe. The regulatory frameworks established by these authorities concerning nanomedicine research generally prioritize critical considerations related to quality and safety—from pharmacological, biodegradation, environmental toxicity, and biocompatibility standpoints.^193^ Investigational medicinal product (IMP) applications are required to state, in addition to the drug development stage, clinical trial phase, duration, study population characteristics, therapeutic use case, and disease specificity. However, as nanocarrier-based drug formulations proceed to clinical trials, their dossiers are also generally required to include the manufacturing process parameters as well as characterization findings to enable a more robust assessment, given that the quality characteristics may not necessarily translate to *in vivo* properties.^194,195^ This also applies in marketing authorizations or new drug applications, where scale-up manufacturing process parametrization must be comprehensively provided in the case of nanomedicines.^196^

While much of regulation existing to guide research and manufacturing of nanocarrier-based pharmaceuticals is derived from interpretations of existing medicine research and development guidelines, Alejandro et al. persuasively argue that there are regulatory challenges that still stem from the lack of standardized definitions of nanomedicine research-related terms that specifically cater to the peculiarities of nanoparticles.^194^ Notably, nanomedicines, when they are offered regulatory definitions, are typically perceived in terms of approximate size limits, which do not sufficiently capture the intricate material properties that may have significant ramifications from a clinical perspective.^194^

Similarly, the Organization for Economic Co-operation and Development (OECD) has steadily released reports that epitomize the evolving regulatory landscape of nanocarriers in the pharmaceutical industry. The ENV/CBC/MONO(2023)­7 is an extensive compilation of national regulatory updates submitted by OECD member states. It consolidates information on policy developments, safety assessment methodologies aligned with the OECD council recommendation, and refinements in best practice frameworks.^197^ A recent version, ENV/CBC/MONO(2024)­1, provides a more recent snapshot, tracking ongoing national initiatives, OECD-endorsed protocols, and considerations for advanced materials as regulatory science progresses. The underlying imperative in these efforts seems to be to establish a coherent, internationally aligned regulatory structure. Nonetheless, disparities currently exist in regional policies, priorities, and directions, complicating a broader effort to bring nanomedicines to the market in a globally integrated manner.

Globally, regulatory agencies, such as the U.S. Food and Drug Administration (FDA) and the European Medicines Agency (EMA), are tasked with establishing frameworks for the evaluation and approval of nanomedicines. As has been emphasized by the authorities, there is an undoubted need for a harmonized international regulatory framework detailing standardized methods for characterizing and assessing nanocarriers’ safety and efficacy. So far, the US and EU have unsurprisingly been at the forefront of regulating nanomedicines and publishing specific guidelines, while other jurisdictions lack clear regulatory direction.

Existing regulations by the FDA and EMA still face some core challenges as nanomedicine rapidly advances. One such challenge is the adequacy of the regulatory framework itself.^198^ The EMA website lists the “*Scientific guidelines on nanomedicines*,” aiming to help developers prepare marketing applications for nanomedicines.^199^ Notably, the EMA’s strategy for regulating nanomedicines involves establishing a dedicated working group to address regulatory issues related to these products.^200^ While this approach enables dynamism in response to new insights and advancements, creating adequate regulations is challenging when knowledge of nanomedicines is limited. This has important implications for maintaining patient safety and regulating the use of nanomedicines in clinical settings.^201^

In a nutshell, nanotechnology is developing faster than regulatory frameworks can keep up. Novel nanomaterials with increased complexity continue to be developed and tuned on the atomic scale, complicating the incorporation of nanocarriers in drug delivery from a regulatory standpoint. Undoubtedly, there needs to be continued collaboration and cross-talk among researchers, industry stakeholders, and regulatory agencies for there to be clear and consistent regulatory directions that ensure the safety and efficacy of these innovative products as they transition to clinical use.

### 4.4 Methodical Challenges in Personalized Medicine

Nanocarriers provide a world of possibilities for the precise control of drug pharmacokinetics and delivery profiles. However, successful translational adoption can only be realized when they can reliably and reproducibly be modified using simple, scalable methods to fit the idiosyncrasies and peculiarities of patients on a case-by-case basis. Currently, this is a challenge because, while the technology appears capable of incorporating patient-derived biomaterials and functionalizations to adapt to patient-specific needs, existing synthesis and fabrication methods of nanomaterials are expensive already as is, and this new requirement would only make them less accessible.

Patient heterogeneity is an important variable that must be accounted for in the design and development of nanocarrier-based drug delivery systems. The tumor microenvironment, for example, often varies from patient to patient and influences the accumulation and distribution of nanoparticles within the tumor tissue. De Maar et al. recommended developing patient-specific formulations tailored to the features of each patient’s tumor microenvironment, accounting for vascular permeability, interstitial fluid pressure, and extracellular matrix composition differences across patients.^202^ From a different perspective, there is the challenge of immune responses and the potential for drug resistance. Some contemporary studies propose integrating multiomics data to develop more precise and effective personalized nanomedicines, considering the complex interplay between genetic, molecular, and environmental factors influencing disease progression and treatment response.^203–205^ These are rather idealistic approaches to further personalizing nanomedicine; and their implications on cost, scalability, and accessibility must be considered.

A machine learning model was recently reported to predict cellular uptake and intracellular trafficking of nanoparticles based on their physicochemical properties and the genetic profile of the target cells.^13,206^ This is an exciting development, and not only could it enable the efficient design of personalized nanoparticle formulations tailored to the specific genetic makeup of individual patients, but it also means that computational advances could democratize access to these tools, making it possible to determine optimal nanocarrier configurations from a library using patient data at minimal cost and within shorter periods than it would ordinarily take to experimentally establish patient fit.

Schematic depictions of the biological barriers that govern pharmacokinetics, nanotechnology applications in personalized medicine, and an overview of theranostic nanocarrier platforms are shown in Figure 4.^12,184,207^

**Figure 4:**
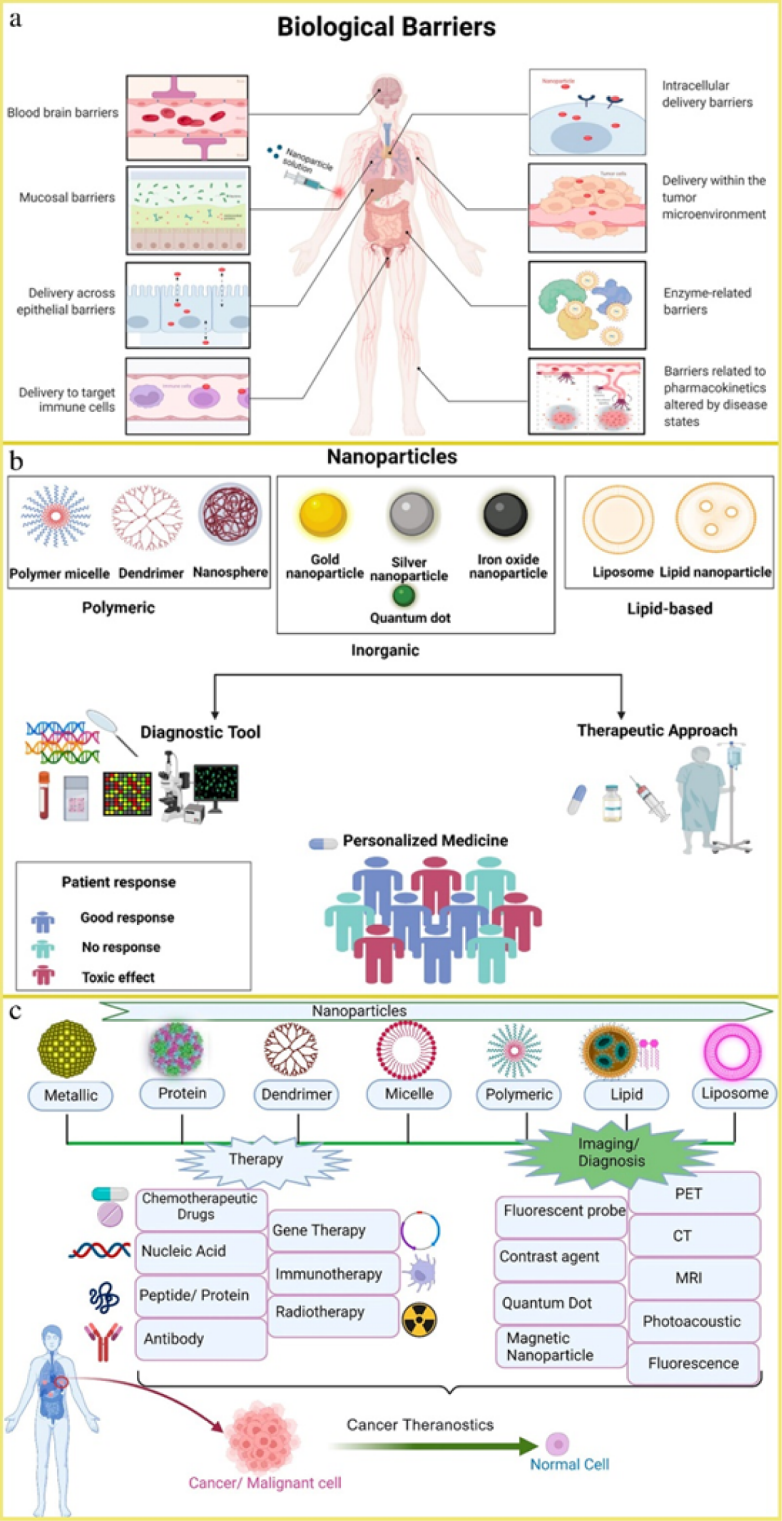
a) Sequence of biological barriers that nanoparticles must overcome for precise drug delivery. Reproduced from ref 184. Available under a CC-BY 4.0 license. Copyright 2022 Waheed et al.; b) A schematic illustration of nanotechnology applications in personalized medicine. Reproduced from ref 207. Available under a CC-BY 4.0 license. Copyright 2022 Alghamdi et al.; c) Nanotheranostic platform for combined therapeutic and diagnostic applications. Reproduced from ref 12. Available under a CC-BY 4.0 license. Copyright 2023 Kashyap et al.

## 5 Conclusion

Nanocarrier-based drug delivery systems show remarkable potential to overcome many limitations of conventional drug delivery methods. The unique properties of nanoparticles, such as size, surface area, and ability to encapsulate and deliver drugs in a controlled and targeted manner, have made them attractive candidates for various therapeutic applications. Nonetheless, nanocarriers are still riddled with important challenges that must be addressed, especially to allow their progress to industrial-scale manufacturing and clinical adoption.

Current pertinent challenges include biocompatibility and toxicity issues arising from the unique interaction of NPs with biosystems and scale-up roadblocks due to technological complexities that impair reproducibility and accessibility. Going forward, the real game-changer in nanocarrier technology will likely come from biocompatible and/or biodegradable NPs that can be produced at scale using cost-effective manufacturing processes; even the most groundbreaking technology will not matter if it is too expensive and inaccessible. Additionally, the global regulatory space appears to be a maze to navigate at the moment, presenting an opportunity for concerted efforts to develop evidence-based policy frameworks that account for near-future advancements in the bionanotechnology field and harmonize global direction to produce standardized protocols for development, characterization, and validation. Therefore, while there will continually be new nanoconstructs being developed and adapted for various disease conditions, future research is likely to drastically increase in areas of sustainable and scalable nanocarrier manufacturing, as well as policy research and regulatory outlooks.

In a nutshell, nanocarrier-based drug delivery is a field that is moving fast, with the potential to completely upend the way diseases are diagnosed and treated. However, it is important to note that none of this happens in a vacuum, and several stakeholders need to commit to collaboration, without which even the most promising breakthroughs in nanocarrier research may fail to make a real difference in patient care.

## Abbreviations

AD: Alzheimer’s Disease
BBB: Blood–Brain Barrier
CBN: Carbon-based nanoparticle
CD: Carbon Dot
CQD: Carbon Quantum Dot
CLEN: Curcumin-Encapsulated Lipidic Nanoconstruct
CNT: Carbon Nanotube
EMA,: European Medicines Agency
EPR: Enhanced Permeability and Retention
FDA: U.S. Food and Drug Administration
GO: Graphene Oxide
HA: Hyaluronic Acid
HD: Huntington’s Disease
HP: High-Pressure Homogenization
LNP: Lipid-based nanoparticle
MRI: Magnetic Resonance Imaging
MRS: Methicillin-Resistant
MSN: Mesoporous Silica Nanoparticle
MWCT: Multiwalled Carbon Nanotube
Nano-Se: Selenium Nanoparticle
NLC: Nanostructured Lipid Carrier
NP: Nanoparticle
OECD: The Organization for Economic Co-operation and Development
OMV: Outer Membrane Vesicle
PEG: Polyethylene glycol
PET: Positron Emission Tomography
PLA: Polylactic Acid
PLGA: Poly­(Lactic-*co*-Glycolic Acid)
PTT: Photothermal Therapy
PVA: Poly­(vinyl alcohol)
ROS: Reactive Oxygen Species
SLN: Solid Lipid Nanoparticle
SWCNT: Single-Walled Carbon Nanotube
